# Draft Genome Sequence of *Klebsiella variicola* Strain KV321 Isolated from Rhizosphere Soil of *Pisolithus tinctorius-Eucalyptus* Mycorrhiza

**DOI:** 10.1128/genomeA.00676-16

**Published:** 2016-07-21

**Authors:** Shao-feng Jiang, Yi Liu, Mi-yun Xiao, Chu-jin Ruan, Zu-jun Lu

**Affiliations:** a College of Life Science, Guangxi Normal University, Guilin Guangxi, China; b Key Laboratory of Ecology of Rare and Endangered Species and Environmental Protection (Ministry of Education) Guilin Guangxi, China; c College of Agriculture, South China Agriculture University, Guangzhou Guangdong, China

## Abstract

The draft genome sequences of *Klebsiella variicola* strain KV321, which was isolated from rhizosphere soil of *Pisolithus tinctorius-Eucalyptus* mycorrhiza, are reported here. The genome sequences contain genes involved in ABC transporter function in multiple-antibiotic drug resistance and colonization. This genomic analysis will help understand the genomic basis of *K. variicola* virulence genes and how the genes play a part in its interaction with other living organisms.

## GENOME ANNOUNCEMENT

The genus *Klebsiella* is a member of the family *Enterobacteriaceae*, whose members range from endophytic nitrogen-fixing to opportunistic pathogens causing various nosocomial and community infections (1–3). *Klebsiella variicola* is one of the *Klebsiella* species. With methods, such as total DNA-DNA hybridization, the monophyly observed in the phylogenetic analysis derived from the sequences of *rpoB*, *gyrA*, *mdh*, *infB*, *phoE*, and *nifH* genes, and the distinct phenotypic traits of clones, Rosenblueth et al. inferred a new species (*Klebsiella variicola*) in the *Klebsiella* genus (4). Since then, *K. variicola* has been isolated and identified from plants (sugarcane, banana, and maize), insects (termites), and hospitalized patients (1–7). *K. variicola* gives rise to cause pneumonia septicemia, meningitis, and urinary tract infections but has different epidemiological dynamics against *Klebsiella pneumoniae* (1, 6, 8). On the other hand, some strains of *K. variicola* are rhizobacteria with the ability to fix nitrogen and promote plant growth (3, 4). Genomic analysis will be helpful to reveal *K. variicola* antibiotic resistance genes and how genes benefit plants by promoting growth.

The draft genome sequences we report were performed on one strain isolated from rhizosphere soil of *Pisolithus tinctorius-Eucalyptus* mycorrhiza with 10-year-old stands of eucalyptus, collected in Dongmen, Fusui county, Chongzuo city, Guangxi Zhuang Autonomous Region. This strain had the highest nitrogen-fixing ability of all strains we separated using Ashby’s N-free medium. Gram staining and Biolog microbial identification showed that strain KV321 was Gram negative and utilized glucose, maltose, sucrose, rhamnose, xylose, arabinose, d-lactose, raffinose, mannose, sorbitol, and inositol but not gelatin. The isolate was identified as *Klebsiella variicola* (SIM 0.610). In addition, antibiotic susceptibility tests demonstrated that *K. variicola* KV321 was resistant to polymyxin, penicillin G, tetracycline, and erythromycin.

Cultivation of *K. variicola* KV321 was performed in Ashby’s N-free medium for 24 h at 30°C under aerobic conditions. Total genomic DNA of the strain was extracted and purified using the DNeasy kit (Tiangen, China), as recommended by the manufacturer. The sequence sequencing and assembly were completed by Zeta Biosciences (Shanghai) Co., Ltd., China, using Illumina HiSeq 2500 with 170× coverage and MicrobeTracker plus version 0.9.1 (incorporates Velvet version 1.2.09). Annotation was added by the NCBI Prokaryotic Genome Annotation Pipeline (2013 release). The whole-genome sequences contain 49 contigs (274 to 773,337 bp length range) after filtering for contigs of >200 bp. The annotation of the genome of *K. variicola* KV321 revealed 5,322 genes, 5,225 coding sequences (CDSs), 75 tRNA genes, six rRNAs, 16 noncoding RNAs (ncRNAs), 58 pseudogenes, and one clustered regularly interspaced short palindrome repeat (CRISPR) array. Bioinformatic analysis showed that most of the predicted genes of *K. variicola* KV321 were involved in a unique combination of virulence, metabolic activity, antibiotic resistance, replication, repair, and biogenesis; also, ABC transporters play a part in multiple-antibiotic resistance genes. Our work will help reveal the genetic elements that participate in the adaptation of *K. variicola* KV321 to the environment of rhizosphere soil of *Pisolithus tinctorius-Eucalyptus* mycorrhiza and the distribution of antibiotic resistance genes.

### Nucleotide sequence accession numbers.

This whole-genome shotgun project has been deposited at DDBJ/ENA/GenBank under the accession no. LQMR00000000. The version described in this paper is version LQMR01000000.
